# Randomized Trial of Fetal Surgery for Moderate Left Diaphragmatic Hernia

**DOI:** 10.1056/NEJMoa2026983

**Published:** 2021-06-08

**Authors:** Jan A. Deprest, Alexandra Benachi, Eduard Gratacos, Kypros H. Nicolaides, Christoph Berg, Nicola Persico, Michael Belfort, Glenn J. Gardener, Yves Ville, Anthony Johnson, Francesco Morini, Mirosław Wielgoś, Ben Van Calster, Philip L.J. DeKoninck

**Affiliations:** Department of Obstetrics and Gynecology, University Hospitals KU Leuven and Academic Department of Development and Regeneration, Biomedical Sciences, KU Leuven, Leuven, Belgium; Institute for Women’s Health, University College London Hospital in london; Hospital Antoine–Béclère, Université Paris–Saclay, Clamart in France; Hospital Clinic and Sant Joan de Déu, Barcelona in London; King’s College Hospital in London; University Hospital Bonn, Bonn, Germany; Hospital Maggiore Policlinico, Milan in Italy; Baylor College of Medicine and Texas Children’s Hospital all in Houston; Mater Mothers’ Hospital, Brisbane, QLD, Australia; all in Houston; Mater Mothers’ Hospital, Brisbane, QLD, Australia; Necker–Enfants Malades Hospital, Paris in France; Children’s Memorial Hermann Hospital all in Houston; Mater Mothers’ Hospital, Brisbane, QLD, Australia; Bambino Gesù Children’s Hospital, Rome in Italy; Medical University of Warsaw, Warsaw, Poland; Academic Department of Development and Regeneration, Biomedical Sciences, KU Leuven, Leuven, Belgium; Department of Obstetrics and Gynecology, University Hospitals KU Leuven Leuven, Belgium and Academic Department of Development and Regeneration, Biomedical Sciences, KU Leuven, Leuven, Belgium; Erasmus MC–University Medical Center Rotterdam, Rotterdam, the Netherlands

## Abstract

**Background:**

Fetoscopic endoluminal tracheal occlusion (FETO) has been associated with increased postnatal survival among infants with severe pulmonary hypoplasia due to isolated congenital diaphragmatic hernia on the left side, but data are lacking to inform its effects in infants with moderate disease.

**Methods:**

In this open-label trial conducted at many centers with experience in FETO and other types of prenatal surgery, we randomly assigned, in a 1:1 ratio, women carrying singleton fetuses with a moderate isolated congenital diaphragmatic hernia on the left side to FETO at 30 to 32 weeks of gestation or expectant care. Both treatments were followed by standardized postnatal care. The primary outcomes were infant survival to discharge from a neonatal intensive care unit (NICU) and survival without oxygen supplementation at 6 months of age.

**Results:**

In an intention-to-treat analysis involving 196 women, 62 of 98 infants in the FETO group (63%) and 49 of 98 infants in the expectant care group (50%) survived to discharge (relative risk, 1.27; 95% confidence interval [CI], 0.99 to 1.63; twosided P = 0.06). At 6 months of age, 53 of 98 infants (54%) in the FETO group and 43 of 98 infants (44%) in the expectant care group were alive without oxygen supplementation (relative risk, 1.23; 95% CI, 0.93 to 1.65). The incidence of pre-term, prelabor rupture of membranes was higher among women in the FETO group than among those in the expectant care group (44% vs. 12%; relative risk, 3.79; 95% CI, 2.13 to 6.91), as was the incidence of preterm birth (64% vs. 22%, respectively; relative risk, 2.86; 95% CI, 1.94 to 4.34), but FETO was not associated with any other serious maternal complications. There were two spontaneous fetal deaths (one in each group) without obvious cause and one neonatal death that was associated with balloon removal.

**Conclusions:**

This trial involving fetuses with moderate congenital diaphragmatic hernia on the left side did not show a significant benefit of FETO performed at 30 to 32 weeks of gestation over expectant care with respect to survival to discharge or the need for oxygen supplementation at 6 months. FETO increased the risks of preterm, prelabor rupture of membranes and preterm birth. (Funded by the European Commission and others; TOTAL ClinicalTrials.gov number, NCT00763737.)

CONGENITAL DIAPHRAGMATIC HERNIA is a surgically correctable defect, but it causes pulmonary hypoplasia, which is characterized by underdeveloped airways and pulmonary vessels.^1^ Congenital diaphragmatic hernia is associated with a high risk of neonatal death and complications, mostly because of pulmonary hypertension and the need for long-term oxygen supplementation.^2,3^

Prenatal congenital diaphragmatic hernia can be classified as severe, moderate, or mild, with expected postnatal survival of approximately 20%, 55%, and 85%, respectively. Expected post-natal survival is based on the quotient of observed-to-expected lung-to-head ratios (i.e., the ratio of the observed lung area to head circumference [measured on ultrasonography] divided by the ratio of that which would be expected in a healthy fetus of the same gestational age) as well as on whether there is intrathoracic herniation of the liver.^4,5^ The quotient of the observed-to-expected lung-to-head ratios is also predictive of early neonatal complications such as the need for supplemental oxygen and of the durations of stay in the neonatal intensive care unit (NICU), use of assisted ventilation, and time to full enteral feeding.^6–8^

Experimental data have shown that prenatal tracheal occlusion attenuates pulmonary hypoplasia^9^ because entrapped lung fluid activates stretch receptors and induces airway proliferation.^10^ Clinically, this technique involves percutaneous fetoscopic endoluminal tracheal occlusion (FETO) with a balloon (video). Prenatal reversal of the occlusion induces maturation.^11,12^ Studies have shown that in fetuses with severe left congenital diaphragmatic hernia, as compared with historical controls, FETO performed at a median gestation of 27 weeks has been associated with greater survival (49% vs. 24%)^13^ and fewer early neonatal respiratory complications,^7,8^ at the expense of an increased risk of prematurity.^14,15^ These studies have also shown a direct relationship between lung size at the time of fetal surgery and later lung growth and survival. In other words, among fetuses with severe pulmonary hypoplasia, those with a higher quotient of the observed-to-expected lung-to-head ratios had a better response to FETO than those with a lower ratio.^16,17^

This finding prompted us to conduct the randomized Tracheal Occlusion to Accelerate Lung Growth (TOTAL) trial (https://www.totaltrial.eu) to compare FETO with expectant prenatal care in women carrying fetuses with moderate congenital diaphragmatic hernia. Both groups received standardized postnatal care.^18,19^ To reduce the risk of very premature birth, FETO was performed at 30 to 32 weeks of gestation.

## Methods

### Trial Design And Participants

This multicenter, open-label, adaptive, parallel-group, superiority trial involving women carrying singleton fetuses with moderate congenital diaphragmatic hernia on the left side was conducted at 12 FETO centers and 46 additional neonatal care centers in Belgium, France, Spain, the United Kingdom, Germany, Italy, Australia, the United States, Poland, Austria, Israel, Switzerland, the Netherlands, the Czech Republic, and Norway (see Table S1 in the Supplementary Appendix, available with the full text of this article at NEJM.org). To participate, the FETO centers had to have performed a minimum of 36 fetoscopies per year, irrespective of the indication; to have performed a minimum of 15 FETO procedures at the time of recruitment; and to have experience with standardized assessment of fetuses with congenital diaphragmatic hernia.^5^

All the women were assessed for eligibility at the FETO centers. The inclusion criteria were a maternal age of 18 years or more, singleton pregnancy, a gestational age at randomization of less than 31 weeks 5 days, congenital diaphragmatic hernia on the left side with no other major structural or chromosomal defects, and moderate pulmonary hypoplasia (defined as the quotient of observed-to-expected lung-to-head ratios of 25.0 to 34.9%, irrespective of liver position, or 35.0 to 44.9% with intrathoracic liver herniation).^4,5^ The exclusion criteria were maternal conditions that would make fetal surgery risky, technical limitations precluding fetal surgery (including those caused by severe maternal obesity, uterine fibroids, or both), an elevated risk of preterm birth (cervical length <15 mm, müllerian anomalies, or placenta previa), and psychological, socioeconomic, or other factors that might prevent adherence to the protocol (available at NEJM.org). We kept a log of eligible nonparticipants and their outcomes.

Eligible women received multidisciplinary counseling and standard information on congenital diaphragmatic hernia and FETO,^20^ as well as information about the concept of a randomized trial.^21^ Fetoscopic placement of a tracheal balloon was carried out at 30 weeks to 31 weeks 6 days of gestation. Reversal of occlusion, either by fetoscopy or by ultrasound-guided puncture of the balloon, was scheduled at 34 weeks 0 days to 34 weeks 6 days of gestation.^22^ The women who were assigned to FETO agreed to live near the FETO center for the duration of tracheal occlusion. If preterm birth was imminent, emergency balloon retrieval was performed in utero (as described above), at the time of delivery while the umbilical cord still connected the infant to the placenta, or by direct puncture immediately after delivery.^23^ After balloon removal, the women were given the option of either delivering in the FETO center or returning home for delivery in their local tertiary referral hospital. In either case, postnatal care was standardized according to international consensus guidelines and was the same for both groups.^18,19^

Approval for the trial was obtained from the relevant research ethics committees and competent authorities in each country. The statistical analysis plan is available with the protocol. The first author vouches for the fidelity of the trial to the protocol and for the accuracy and completeness of the data.

### Randomization

After assessment for eligibility, the women were randomly assigned, in a 1:1 ratio, to one of the two treatment groups, without stratification factors. Randomization was performed by a fetal medicine specialist using a purposely developed secure website. Block randomization was used for an equal distribution per group at every analysis (a single block for each analysis). The randomization sequence was generated by the statistician.

### Outcome Measures

The initial primary outcome was survival to discharge without bronchopulmonary dysplasia.^24^ At the prespecified administrative review, while the data were still blinded, the data monitoring and safety committee redefined the primary outcome as survival to discharge from the NICU. The committee also expanded the time point for the need for oxygen supplementation to 6 months; survival to 6 months without oxygen supplementation became one of the two primary outcome measures. Oxygen supplementation was defined as any need for additional oxygen for respiratory support delivered by nasal cannula, high-flow devices, continuous positive airway pressure ventilation, or mechanical ventilation. The secondary and exploratory outcomes included surgical and pregnancy complications, fetal and neonatal survival, and complications in early infancy (Table S2).

### Statistical Analysis

Our trial had a group-sequential design and five interim analyses to enable early stopping for clear superiority, with a two-sided alpha level of 5% with an O’Brien–Fleming alpha-spending function^25^ and a power of 80%. Assuming that survival to discharge that was 20 percentage points higher in the FETO group than in the expectant care group would be highly relevant, 98 participants per group would be required if the trial was not discontinued early. The design could lead to a statistically significant result with an observed absolute increase of approximately 15 percentage points in survival to discharge, which was still considered to be clinically relevant. No formal boundaries for futility were considered. Details on sample-size considerations for the two primary outcomes and other details are provided in the statistical analysis plan, which is available with the protocol.

We analyzed the two primary outcomes using the z test for proportions according to the intention-to-treat principle. To control for multiplicity, significance testing for the two primary outcomes was performed only if the result for survival to discharge was statistically significant. A secondary analysis was performed according to the per-protocol principle. Secondary outcomes were analyzed only according to the intention-to-treat principle, without formal significance testing. We report relative risks, differences in percentages, and differences in medians with 95% confidence intervals because there was no adjustment for multiplicity in the analyses of secondary and exploratory outcomes. These confidence intervals should not be used to infer definitive treatment effects. Safety outcomes and adverse events are reported descriptively.

## Results

### Trial Participants

**Figure 1:**
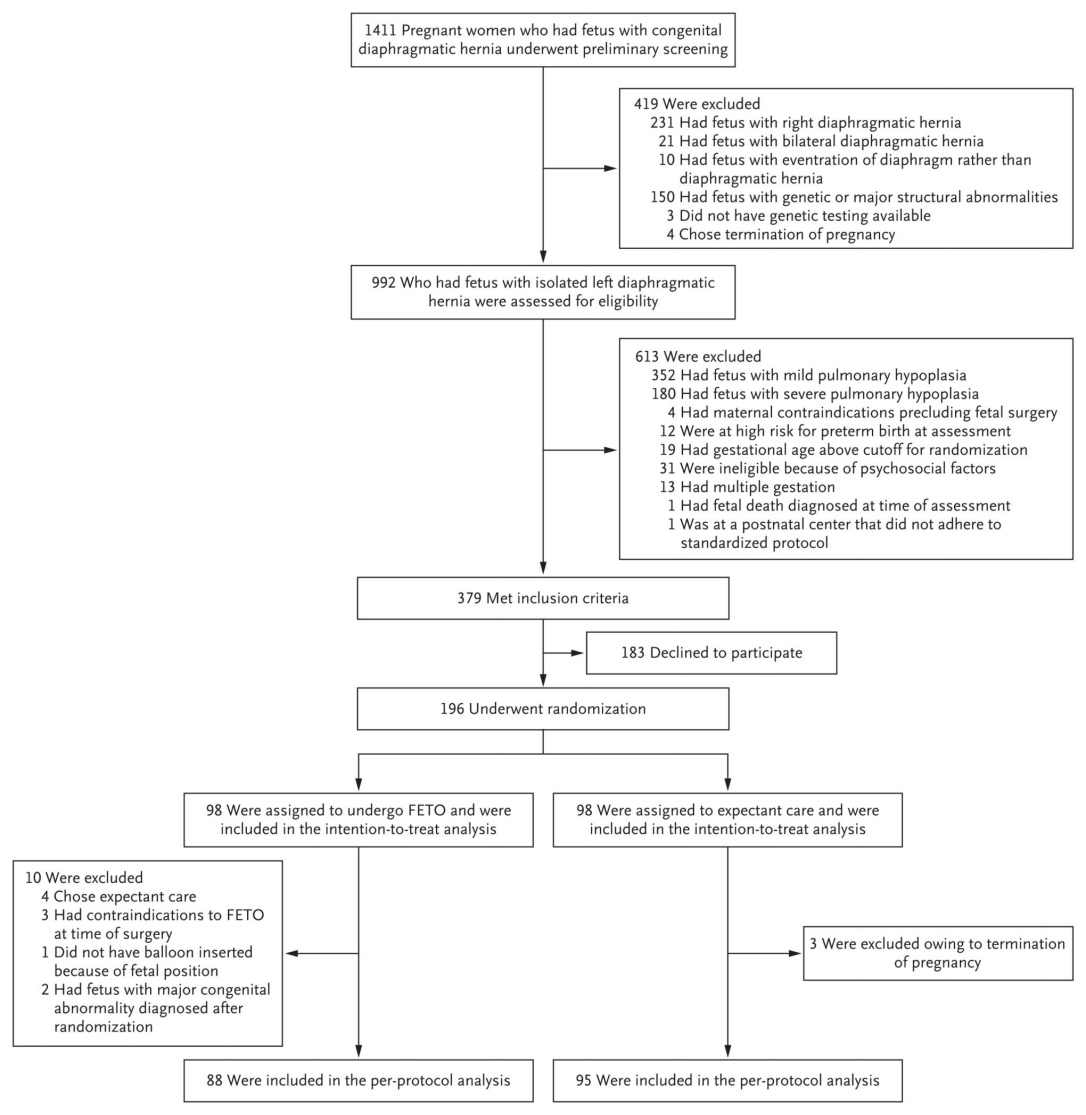
Screening, Randomization, and Analysis. Among the women who were excluded from the fetoscopic endoluminal tracheal occlusion (FETO) group after inclusion in the intention-to-treat analysis, four chose expectant care and three had contraindications to FETO (one had a short cervix, one had spontaneous membrane rupture, and in one there was an unfavorable fetal position before FETO was attempted). In another participant who was excluded from the FETO group, the fetal mouth was not accessible after trocar insertion into the amniotic cavity, and a balloon was not inserted. Two fetuses had major congenital abnormalities that were diagnosed after randomization (Simpson–Golabi–Behmel syndrome in one fetus and a mutation in the gene encoding filamin A [*FLNA*] in the other).

Recruitment started in October 2008 and was completed in May 2019. A total of 1411 women carrying fetuses with congenital diaphragmatic hernia underwent preliminary assessment, and 379 met the inclusion criteria; of these women, 196 (51.7%) provided written informed consent to participate and were randomly assigned to FETO (98 women) or expectant care (98 women) (Fig. 1). None of the participants withdrew consent or were lost to follow-up; thus, all the participants were included in the intention-to-treat analysis. This intention-to-treat analysis included 6 cases of genetic, syndromic, or severe structural abnormalities in the FETO group and 3 cases in the expectant care group. Some of these abnormalities, which were diagnosed either before or after birth, led to exclusion from the per-protocol analysis (details are provided in Table S3). In the FETO group, 10 women in the intention-to-treat analysis were excluded from the per-protocol analysis, including 8 who did not undergo FETO but received expectant care, and 2 who had infants in whom there was a postnatal diagnosis of a genetic abnormality for which postnatal palliative care was warranted (details are provided in Fig. 1). In the expectant care group, 3 participants did not receive the assigned intervention because they chose pregnancy termination. Therefore, 88 participants in the FETO group and 95 participants in the expectant care group were included in the per-protocol analysis (Fig. 1).

The baseline characteristics of the participants were similar in the two groups (Table 1). In the FETO group, the procedure was carried out in 91 of 98 of the women (93%) who had undergone randomization, and a balloon was successfully inserted in the fetal trachea in all but 1 woman (Table S4). In 3 fetuses, spontaneous balloon deflation was first observed at 31 weeks 0 days of gestation, 32 weeks 3 days of gestation, and 32 weeks 4 days of gestation, respectively. In 1 fetus, a second balloon was inserted at 33 weeks 1 day, and this balloon also spontaneously deflated. In 54 of the 90 women in whom the balloon was successfully inserted (60%), the balloon was removed as originally planned, whereas in 35 women (39%), balloon removal was undertaken earlier, mainly because the women went into spontaneous labor or had preterm, prelabor rupture of membranes; in 1 woman (1%), no removal was attempted because spontaneous deflation occurred. Balloon removal was mostly performed by means of fetoscopy (in 98% of elective removals and 69% of emergency procedures). In all but 1 fetus, reversal of occlusion was successful.

### Primary Outcomes

The trial was not stopped early for superiority. The percentages of infants who survived to discharge from the NICU were 63% (62 of 98 infants) in the FETO group and 50% (49 of 98 infants) in the expectant care group (relative risk, 1.27; 95% confidence interval [CI], 0.99 to 1.63; two-sided P = 0.06) (Table 2). The percentages of infants who survived without oxygen supplementation at 6 months of age were 54% (53 of 98 infants) and 44% (43 of 98 infants), respectively (relative risk, 1.23; 95% CI, 0.93 to 1.65) (Table 2). The per-protocol analysis yielded similar results with respect to survival to discharge from the NICU (relative risk, 1.23; 95% CI, 0.96 to 1.59) and survival at 6 months of age without oxygen supplementation (relative risk, 1.21; 95% CI, 0.90 to 1.62).

### Secondary And Exploratory Outcomes

Two unexplained fetal deaths occurred, one in the FETO group at 37 weeks 2 days of gestation and one in the expectant care group at 36 weeks 2 days of gestation. The incidence of preterm, prelabor rupture of membranes was 44% in the FETO group and 12% in the expectant care group (relative risk, 3.79; 95% CI, 2.13 to 6.91); the respective incidences of preterm birth were 64% and 22% (relative risk, 2.86; 95% CI, 1.94 to 4.34). The median gestational age at delivery was approximately 2 weeks earlier in the FETO group than in the expectant care group (Table 2).

Results in infants who survived to NICU discharge are shown in Table S5; these results are presented descriptively. However, there were no obvious between-group differences in the duration of NICU stay or neonatal complications. The outcomes and characteristics of 183 eligible participants who did not undergo randomization are shown in Figure S1.

### AdverseEveNTS

Serious and other adverse events are reported in Table 3. Preterm, prelabor rupture of membranes and preterm delivery were the most frequent adverse events in the FETO group. No cases of placental abruption occurred in either group, and no intraoperative complications occurred in the FETO group.

There were two problematic balloon removals. One infant could not be resuscitated after an emergency attempted removal of the balloon with an endoscope at the time of delivery while the umbilical cord still connected the infant to the placenta. The balloon, which was thought to have been punctured, was found intact within the trachea at the postmortem examination. In retrospect, the airways had been explored under direct vision, so the operator had less control of the procedure and less adequate assessment of the position and status of the balloon than would have been provided with endoscopic vision. In one other infant, postnatal removal of the balloon took up to 3 minutes from the time of birth until intubation.

In the FETO group, tracheomalacia was diagnosed in one infant at 2 months of age and tracheomalacia was suspected in another infant. Details are provided in Table 3.

## Discussion

In this multicenter, randomized trial involving woman carrying singleton fetuses with isolated moderate congenital diaphragmatic hernia on the left side, the chance of survival of infants to discharge from the NICU or survival without oxygen supplementation at 6 months of age was not significantly greater with prenatal intervention with FETO at 30 to 32 weeks of gestation than with expectant care. The confidence interval is compatible with an increase in survival to discharge from the NICU that is 1 percentage point lower to 63% percentage points higher in the FETO group than in the expectant care group. The risk of preterm, prelabor rupture of membranes was 3.8 times as high and the risk of preterm birth was 2.9 times as high in the FETO group as in the expectant care group. No other serious complications occurred in the women, and there were no obvious between-group differences in the duration of neonatal intensive care or the duration of ventilatory support in the infants. However, the trial was not powered for these or other complications associated with prematurity.

The results in the expectant care group in this trial are consistent with those in contemporary nonintervention studies involving prenatally diagnosed congenital diaphragmatic hernia,^26^ with similar fetal lung measurement methods and protocols for postnatal care.^27,28^ Little was known before this trial about outcomes of FETO in fetuses with moderate pulmonary hypoplasia, because nearly all previous studies were limited to fetuses with severe pulmonary hypoplasia.^14,15,29^

A companion article now published in the *Journal* describes a randomized trial involving fetuses with isolated congenital diaphragmatic hernia and severe pulmonary hypoplasia on the left side.^30^ In that trial, which we carried out in parallel to this trial and in many of the same centers, we found that 40% of the infants of mothers assigned to FETO, as compared with 15% of those in the expectant care group, survived to discharge from the NICU (relative risk, 2.67; 95% CI, 1.22 to 6.11).^30^ In addition to the between-trial difference in the severity of congenital diaphragmatic hernia, the timing of FETO also differed (i.e., 27 to 29 weeks of gestation in the trial of severe pulmonary hypoplasia and 30 to 32 weeks of gestation in this trial). It is possible that the delay in tracheal occlusion contributed to the lack of significant improvement in survival in the current trial. The main reason for such delay was to minimize the risk of procedure-related preterm, prelabor rupture of membranes and very preterm birth in infants with moderate disease, given the considerably lower risk of death among these infants than among those with severe disease and the serious consequences of extreme prematurity associated with congenital diaphragmatic hernia.^31^

A limitation of the current trial is the long time period required to complete it,^32^ during which the protocols for postnatal care of congenital diaphragmatic hernia may have changed^33–35^; however, this would apply to both trial groups. Data are not available on medium-term or long-term outcomes, and the trial was not powered to inform uncommon fetal, pediatric, and maternal complications. It is difficult to compare our results, which were obtained in fetuses with pre-natally diagnosed congenital diaphragmatic hernia, with those reported by centers that have a different case mix (i.e., those that typically care for women referred late in pregnancy or infants transferred after birth).^33,34^ In addition, although the investigators were aware of the treatment group assignments, we do not think this would have affected the care of the patients. Also, the performance of FETO requires a wide range of skills, from the ability to perform ultrasound-guided needle procedures to expertise in advanced fetoscopy, as well as the round-the-clock availability of staff members with those skills, and our trial centers had high caseloads. Therefore, the findings should not be generalized to less experienced centers.^23^

Future studies are needed to assess potential strategies to reduce FETO-associated complications — such as the use of thinner fetoscopic instruments to reduce the risk of preterm, prelabor rupture of membranes,^36,37^ ultrasound-guided puncture rather than fetoscopy for removal of the balloon,^23^ and the use of a balloon with a magnetic valve that can be opened noninvasively.^38,39^ Data are also lacking to assess whether a longer occlusion period would result in additional lung growth.^16,40^ Finally, a post hoc analysis of the combined data from this trial and the companion trial involving fetuses with severe pulmonary hypoplasia,^30^ with the use of the quotient of the observed-to-expected lung-to-head ratios as a continuous variable, may help to inform inclusion criteria for further studies involving fetuses with moderate pulmonary hypoplasia.

This randomized trial involving fetuses with isolated moderate congenital diaphragmatic hernia on the left side did not show a significant increase in survival of infants to NICU discharge or a reduction in the need for oxygen supplementation at 6 months of life among infants assigned to FETO. FETO resulted in increased risks of preterm, prelabor rupture of membranes and preterm birth.

## Supplementary Material

Supplementary appendix

## Figures and Tables

**Table 1:** Baseline Characteristics of the Mothers and Infants.^*^

Characteristic	FETO Group (N = 98)	Expectant Care Group (N = 98)
Median maternal age (IQR) — yr	31.1 (27.5–33.7)	31.6 (27.4–34.9)
Median gestational age at randomization (IQR) — wk	28.4 (26.6–29.6)	28.2 (27.0–29.3)
Nulliparous women — no. (%)	45 (46)	48 (49)
Median BMI (IQR)^†^	23.8 (20.4–28.2)	22.2 (21.0–25.9)
Cigarette smoker — no. (%)	11 (11)	8 (8)
Alcohol use during pregnancy — no. (%)	1 (1)	1 (1)
Race — no. (%)^‡^		
White	87 (89)	88 (90)
Asian	3 (3)	3 (3)
Black	2 (2)	2 (2)
Other	6 (6)	5 (5)
Findings on ultrasonography at randomization		
Median quotient of observed-to-expected lung-to-head ratios (IQR) — %^§^	30.9 (28.0–34.0)	31.0 (28.0–34.5)
Intrathoracic liver herniation — no. (%)	79 (81)	78 (80)
Median deepest vertical pocket of amniotic fluid (IQR) — cm	6.0 (5.0–7.6)	6.4 (5.5–7.7)
Median cervical length (IQR) — mm	37 (33–40)	36 (31–40)
Placental position — no. (%)		
Anterior	50 (51)	47 (48)
Posterior	43 (44)	47 (48)
Fundal	5 (5)	4 (4)

* FETO denotes fetoscopic endoluminal tracheal occlusion, and IQR interquartile range.

† The body-mass index (BMI) is the weight in kilograms divided by the square of the height in meters.

‡ Race was reported by the participants.

§ The quotient of observed-to-expected lung-to-head ratios is calculated as the ratio of the ultrasonographic measurement of the observed lung area to head circumference (measured on ultrasonography) divided by the ratio of that which would be expected in a healthy fetus of the same gestational age.

**Table 2:** Outcomes According to Trial Group in the Intention-to-Treat Population.^*^

Outcome	FETO Group (N=98)	Expectant Care Group (N=98)	Relative Risk (95%Cl)	Difference (95%Cl)^†^
**Primary outcomes — no. (%)**				
Survival to discharge from NICU	62 (63)	49 (50)	1.27 (0.99 to 1.63)	13 (−l to 28)^‡^
Survival to 6 mo without oxygen supplementation	53 (54)	43 (44)	1.23 (0.93 to 1.65)	10 (−4 to 25)
**Secondary and exploratory outcomes**				
Postnatal survival — no. (%)				
To 28 days^§^	67 (68)	56 (57)	1.20 (0.96 to 1.50)	11 (−2 to 25)
To 56 days^§^	65 (66)	54 (55)	1.20 (0.96 to 1.52)	11 (−3 to 26)
To 6 mo	61 (62)	49 (50)	1.24 (0.97 to 1.61)	12 (−2 to 27)
Preterm, prelabor rupture of membranes¶				
Median gestational age (1QR) — wk^§^	34.0 (33.0 to 35.0)	34.6 (31.4 to 35.6)‖		0.6 (−2.0 to 3.3)
Rupture of membranes at <37 wk— no./total no. (%)^§^	43/97 (44)	11/93 (12)^**^	3.79 (2.13 to 6.91)	33 (21 to 46)
Rupture of membranes at <34 wk— no./total no. (%)^§^	21/97 (22)	5/93 (5)^**^	4.07 (1.68 to 10.1)	16 (6 to 27)
Gestational age at birth^§¶^				
Median gestational age (IQR) — wk	35.9 (34.3 to 37.9)	38.1 (37.0 to 38.9)		−2.3 (−3.0 to −1.5)
<37 wk — no./total no. (%)	62/97 (64)	21/94 (22)	2.86 (1.94 to 4.34)	42 (29 to 56)
<34 wk — no./total no. (%)	19/97 (20)	7/94 (7)	2.63 (1.20 to 5.89)	12 (2 to 23)
<32 wk — no./total no. (%)	6/97 (6)	3/94 (3)	1.94 (0.55 to 6.93)	3 (−5 to 10)
Placental abruption — no./total no. (%)^§¶^	0/97	0/94	NC^‡^	0 (−5 to 5)
Neonatal outcomes in live births^¶^				
Median birth weight (IQR) — g^§^	2500 (2200 to 2855)	2945 (2500 to 3292)		−445 (−635 to −260)
Neonatal repair of defect— no./total no. (%)	81/97 (84)	70/94 (74)	1.12 (0.97 to 1.31)	9 (−3 to 21)
Use of prosthetic patch for repair— no./total no. (%)	67/81 (83)	50/70 (71)	1.16 (0.97 to 1.41)	11 (−3 to 26)
Median time to repair of defect (IQR) — days	3 (2 to 6)	3 (2 to 5)		0 (0 to 2)
ECMO — no./total no. (%)	20/97 (21)	19/94 (20)	1.02 (0.59 to 1.78)	0 (−12 to 13)

* Data are for the overall intention-to-treat-population unless otherwise specified. The between-group differences may not be the expected values because of rounding. ECMO denotes extracorporeal membrane oxygenation, NC not calculated, and NICU neonatal intensive care unit.

† Differences were calculated as the absolute difference in percentages (expressed in percentage points) for dichotomous data or as the difference in medians for continuous data.

‡ P=0.06 for the comparison between the FETO group and the expectant care group.

§ This was an exploratory outcome.

¶ One spontaneous fetal death in each group and three terminated pregnancies in the expectant care group were excluded.

‖ In addition to the one spontaneous fetal death and three terminated pregnancies, data on two additional pregnancies were missing. For the calculation of relative risk and the difference in medians, missing values were addressed according to the protocol.

** In addition to the one spontaneous fetal death and three terminated pregnancies, data on one additional pregnancy were missing. For the calculation of relative risk and the difference in percentages, missing values were addressed according to the protocol.

‡ The relative risk was not calculated because both percentages equal 0.

**Table 3:** Adverse Events in the Safety Population.*

Event	FETO Group (N = 91)^†^	Expectant Care Group (N = 95)^‡^
	number/total number (%)	
**Serious adverse events**		
Fetal death		
<24 hr after FETO	0/91	NA
Any time during pregnancy^§^	1/91 (1)	1/95 (1)
Placental abruption		
<24 hr after FETO	0/91	NA
Any time during pregnancy^§^	0/91	0/95
Lengthy balloon removal procedure^¶^	1/91 (1)	NA
Severe preeclampsia‖	1/91 (1)	0/95
Chorioamnionitis‖	1/91 (1)	1/95 (1)
Abnormal cardiotocographic findings before labor‖	3/91 (3)	2/95 (2)
Intrauterine growth restriction‖	2/91 (2)	3/95 (3)
Preterm, prelabor rupture of membranes <37 wk	42/91 (46)	11/94 (12)
Delivery <37 wk	60/91 (66)	22/95 (23)
Neonatal death due to failure of balloon removal	1/91 (1)	NA
Perinatal asphyxia, umbilical pH <7.00	0/70	2/63 (3)
ECMO	18/91 (20)	19/95 (20)
Conditions in infants who survived to discharge		
Bronchopulmonary dysplasia	37/57 (65)	32/49 (65)
Pulmonary hypertension	42/57 (74)	33/49 (67)
Periventricular leukomalacia	3/57 (5)	1/49 (2)
Sepsis	19/57 (33)	17/49 (35)
Necrotizing enterocolitis	2/57 (4)	0/49
Intraventricular hemorrhage >grade III	0/57	0/49
Retinopathy of prematurity	0/57	0/49
Death		
Neonatal, <28 days	28/91 (31)	38/95 (40)
Between 28 days and 6 mo	6/91 (7)	7/95 (7)
Tracheomalacia or tracheal changes^**^	2/91 (2)	NA
**Other adverse events**		
Fetal hydrops‖	0/91	1/95 (1)
Bilateral fetal hydrothorax‖	1/91 (1)	0/95
Chorioamniotic membrane separation	23/88 (26)	NM
Vaginal bleeding‖	3/91 (3)	0/95
Bleeding resulting from trocar insertion during fetoscopy‖	5/91 (5)	NA
Polyhydramnios first manifesting at follow-up ultrasonographic examination	22/88 (25)	NM
Gastroesophageal reflux in infants who survived to discharge	30/55 (55)	19/39 (49)

* The safety population included all the participants who underwent randomization and who effectively received their assigned prenatal treatment (see the statistical analysis plan, which is available with the protocol). NA denotes not applicable, and NM not measured.

† This group includes the 98 participants who were randomly assigned to undergo FETO minus 7 participants who did not have a surgical procedure: 4 participants decided not to undergo FETO, and the other 3 did not undergo FETO owing to ruptured membranes (1 participant), a very short cervix (1 participant), and poor fetal position precluding an attempt to insert the trocar (1 participant).

‡ This group includes the 98 participants who were randomly assigned to receive expectant care minus 3 participants who opted for termination of pregnancy.

§ In the FETO group, a postmortem examination did not identify any plausible cause for the intrauterine fetal death. In the expectant care group, a placental examination showed a massive thrombosis with complete vascular occlusion in one of the umbilical arteries. Examination of the mother’s blood showed a low level of protein S and low positivity for lupus anticoagulant. The data and safety monitoring board considered these findings insufficient to determine causation and categorized the death as “unexplained.”

¶ Because of unavailability of the maternal fetal medicine specialist, balloon puncture was performed after vaginal birth by the attending neonatologist. The neonatal umbilical artery pH was 7.34, and conventional mechanical ventilation was promptly initiated. The infant died of acute bilateral pneumonia at 23 days.

‖ The occurrence or absence of anticipated adverse events was indicated in check boxes (yes or no). Any other event that was not anticipated could be reported in a free-text field. For the events indicated in check boxes, the denominator takes into account missing values.

** Tracheomalacia was suspected in 1 infant who was discharged without respiratory support. Inflammation and scarring were observed during bronchoscopy at the time of fundoplication at 5 months of age; follow-up bronchoscopy at 10.5 months of age showed that these conditions had improved. The infant had known reflux, which was considered to be a potential cause of the findings, and thus the infant was considered to have possible tracheomalacia.
