# Supplementary material for: Randomized Trial of Fetal Surgery for Moderate Left Diaphragmatic Hernia
Source: N Engl J Med. Author manuscript; Available in PMC 2022 Aug 28. (PMC7613454; doi:10.1056/NEJMoa2026983)
Supplement: Supplementary appendix [file EMS152843-supplement-Supplementary_appendix.pdf]

Supplementary Appendix

This appendix has been provided by the authors to give readers additional information about their work.

Supplement to: Deprest JA, Benachi A, Gratacos E, et al. Randomized trial of fetal surgery for moderate left diaphragmatic hernia. N Engl J Med 2021;384:119-29. DOI: 10.1056/NEJMoa2026983

TOTAL trial for moderate hypoplasia

Supplementary appendix

Table of contents of the appendix

List of investigators		2
Figure S1	Outcomes of eligible non-participants	6
Supplementary Table S1	Alphabetical list of FETO and neonatal management centers	7
Supplementary Table S2	List of reported outcomes and safety endpoints	9
Supplementary Table S3	Associated anomalies diagnosed after randomization	12
Supplementary Table S4	Operative outcomes in patients in the FETO arm	13
Supplementary Table S5	Secondary neonatal outcomes in survivors to discharge	14
Supplementary Table S6	Interim analyses	15
Supplementary Table S7	Case load and historical outcomes for postnatal management centers	16

List of investigators

Principal Investigators

Jan A Deprest[1] M.D. Ph.D., Alexandra Benachi[2] M.D. Ph.D., Eduard Gratacos[3] M.D. Ph.D., Kypros H Nicolaides[4] M.D., Christoph Berg[5] M.D. Ph.D., Nicola Persico[6] M.D. Ph.D., Michael Belfort[7] M.D. Ph.D., Glenn J. Gardener[8] M.D. Ph.D., Yves Ville[9] M.D. Ph.D., Anthony Johnson[10] M.D., Francesco Morini[11] M.D. Ph.D., Miroslaw Wielgos[12] M.D. Ph.D., Ben Van Calster[1] Ph.D., Philip LJ DeKoninck[1, 17] M.D. Ph.D.

Data Monitoring and Safety Committee and data managers

Paul Lewi †[1], Ph.D., Tim Van Mieghem[1,13] M.D. Ph.D., Francesca Russo[1] M.D. Ph.D., Hugo Devlieger[1] M.D. Ph.D., Michael Harrison[14] M.D. Ph.D., Francois I Luks[15] M.D. Ph.D., Angélique Rezer[1] LL.M., Kris Dierickx[1] Ph.D., Beverley Power[16]

External committee drafting neonatal guidelines

Dick Tibboel[17] M.D. Ph.D., Thomas Schaible[18] M.D. Ph.D., Laurent Storme[19] M.D. Ph.D.

Affiliations

[1] The University Hospitals KU Leuven, Leuven, Belgium; [2] Hospital Antoine Béclère, Université Paris Saclay, Clamart, France; [3] Hospital Clinic and Sant Joan de Deu, Barcelona, Spain; [4] King's College Hospital, London, UK; [5] University Hospital Bonn, Bonn, Germany; [6] Hospital Maggiore Policlinico, Milano, Italy; [7] Baylor College of Medicine and Texas Children's Hospital, Houston, TX, USA; [8] Mater Mother's Hospital, Brisbane, Australia; [9] Hospital Necker, Paris, France; [10] Children's Hermann Memorial Hospital, Houston, TX, USA; [11] Bambino Gesù Children's Hospital, Rome, Italy; [12] Medical University of Warsaw, Warsaw, Poland; [13] Mount Sinai Hospital, Toronto, ON, Canada; [14] University of California at San Francisco, CA, USA; [15] Hasbro Children's Hospital, Providence, RI, USA; [16] C.D.H. UK Parent Organization, London, UK; [17] Erasmus MC University Medical Center Rotterdam, Rotterdam, The Netherlands; [18] Mannheim University Hospital, Mannheim, Germany; [19] University Medical Center Lille, Lille, France.

† Prof P. Lewi deceased during the trial

Other TOTAL trial for moderate CDH collaborators

The University Hospitals KU Leuven, Leuven, Belgium – Liesbeth Lewi M.D. Ph.D., Roland Devlieger M.D Ph.D., Anne Debeer, M.D. Ph.D., Herbert De Caluwe M.D. Ph.D.; **Hôpital Bicetre, Paris France** – Anne-Gael Cordier M.D. Ph.D., Nolwenn Le Sache M.D., Virginie Fouquet M.D.; **Brugmann Hospital, Brussels, Belgium** – Jacques Jani M.D. Ph.D.; **Hospital**

Clinic/Hospital Sant Joan de Deu, Barcelona – Josep Maria Martinez M.D., Olga Gomez M.D., Rogelio Cruz-Martinez M.D., Africa Pertierra M.D.; **King's College Hospital, London, UK** - Ramona Cazacu M.D., Theodore Dassios M.D., Shailesh Patel M.D., Ewelina Litwinska M.D.; **Universitätsklinikum Bonn, Bonn, Germany** – Brigitte Strizek M.D., Andreas Müller M.D., Andreas Heydweiller M.D.; **Ospedale Maggiore Policlinico, Milan, Italy** – Isabella Fabietti M.D., Fabio Mosca M.D., Ernesto Leva M.D.; **Baylor College of Medicine/Texas Children's Hospital, Houston, USA** – Alireza A. Shamshirsaz M.D., Joseph A. Garcia-Prats M.D., Timothy Lee M.D.; **Mater Mothers Hospital, Brisbane, Australia** – Scott Petersen M.D., Lucy Cooke M.D., Craig Mcbride M.D.; **Hôpital Necker – Enfants Malades, Paris, France** – Julien Stirneman M.D., Elsa Kermorvant-Duchemin M.D., Naziha Ken-Dunlop M.D.; **University of Texas Health Science Center / Children's Memorial Hermann, Houston, USA** – Mary Austin M.D., Suzanne M. Lopez M.D., Kuojen Tsao M.D.; **Bambino Gesù Children's Hospital, Rome, Italy** – Anita Romiti M.D., Irma Capolupo M.D., Laura Valfre M.D.; 1**st Department of Obstetrics and Gynecology, Medical University of Warsaw, Poland** – Przemyslaw Kosinski M.D., Pawel Krajewski M.D., Andrzej Kaminski M.D.; **Radboudumc/Amalia Children's Hospital, Nijmegen, The Netherlands** – Mallory Woiski M.D., Willem P. de Boode M.D., Horst Daniels-Scharbatke M.D.; **Hôpital Robert Debré, Paris, France –** Jonathan Rosenblatt M.D.; Michael Levy M.D., Elisabeth Carricaburu M.D.; **Hôpital Bretonneau, Tours, France** – Franck Perrotin M.D., Antoine Bouissou M.D., Hubert Lardy M.D., **Erasmus MC University Medical Center, Rotterdam, The Netherlands** – Alex J. Eggink M.D., Rene M.H. Wijnen M.D.; **CHU de Bordeaux, Bordeaux, France** – Loic Sentilhes M.D., Olivier Brissaud M.D., Frederic Lavrand M.D.; **LKH-Universitäts Klinikum Graz, Graz, Austria** – Philipp Klaritsch M.D., Berndt Urlesberger M.D., Holger Till M.D.; **Hôpital Trousseau, Paris, France** – Jean-Marie Jouannic M.D., Julia Guilbert M.D., Sabine Irtan, M.D.; **CHU Amiens, Amiens, France** – Arthur Foulon M.D., Pierre Tourneux M.D., Philippe Buisson M.D.; **Saint Luc UCL, Brussels, Belgium** – Jean-Marc Biard M.D., Catheline Hocq M.D., Catherine De Magnée M.D.; **CHU Caen, Caen, France –** Guillaume Benoist M.D., Valerie Datin-Dorriere M.D.; Thierry Petit M.D.; **CHU de Dijon, Dijon, France** – Thierry Rousseau M.D., Stephanie Litzler-Renault M.D., Emmanuel Sapin M.D.; **Medical University of Gdansk, Gdansk, Poland** – Krzysztof Preis M.D., Iwona Janczewska M.D., Andrzej Golebiewski M.D.; **CHRU Lille, Lille, France –** Veronique Houfflin-Debarge M.D., Dyuti Sharma M.D.; **Gregorio Maranon Hospital, Madrid, Spain** – Eugenia Antolin Alvarado M.D., Belen Bernardo Atienza M.D., Juan Carlos de Agustín Asensio M.D.; **The Royal Women's Hospital, Melbourne, Australia** – Stefan Kane M.D., Rodney Hunt M.D.; **Women's and Children's Hospital, Adelaide, Australia** – Peter Muller M.D., Jodie Dodd M.D., Catherine Cord-Udy M.D.; **University of Michigan Health System, Ann Arbor, USA** – Erin E. Perrone M.D.; **Chelsea and Westminster Hospital NHS Foundation Trust, London, UK** – Makrina

Savvidou M.D., Shu-ling Chuang M.D., Simon Clarke M.D.; **CHU Clermont-Ferrand, Clermont-Ferrand, France** – Amelie Delabaere M.D., Karen Coste M.D., Maguelonne Pons M.D.; **Universitätsklinik Hamburg-Eppendorf, Hamburg, Germany** – Kurt Hecher M.D. Ph.D.; **St George's University Hospitals, London, UK** – Basky Thilaganathan M.D. Ph.D., Nigel Kennea M.D., Bruce Okoye M.D.; **University Hospital Mannheim, Mannheim, Germany** – Katrin Zahn M.D., Christiane Otto M.D.; **CHU Montpellier, Montpellier, France** – Florent Fuchs M.D., Odile Pidoux M.D., Dominique Forgues M.D.; **CHU Nancy, Nancy, France** – Alexis Maatouk M.D., Mahmoud Rouabah M.D., Jean-Louis Lemelle M.D.; **The Institute for the Care of Mother and Child, Prague, Czech Republic** – Ladislav Krofta M.D., Zbynek Stranak M.D., Michal Rygl M.D.; **CHU Rennes, Rennes, France** – Gwenaelle Le Bouar M.D., Alain Beuchée M.D., Alexis Arnaud M.D.; **CHU Saint Etienne, Saint Etienne, France** – Marie-Noelle Varlet M.D., Caroline Paricio M.D., Fabienne Prieur M.D.; **St Olavs Hospital, Trondheim, Norwich** – Brigitte Heiberg Kahrs M.D., Øystein Drivenes M.D.; **University Hospital Zurich, Zurich, Suisse** – Nicole Ochsenbein-Kölble M.D., Ueli Möhrlen M.D.; **Medway Maritime Hospitals, Gillingham, UK** – Ranjit Akolekar M.D., Shakuntala Kalla M.D.; **UZ Gent, Gent, Belgium** – Elke Sleurs M.D.; **Royal Free London NHS Foundation Trust, London, UK** – Rezan Abdul-Kadir M.D.; **Baylor College of Medicine, Houston, USA** – Karolina Adam M.D.; **Hospital Universitario de Canarias, Tenerife, Spain** – Margarita Alvarez de la Rosa M.D.; **Covenant Medical Group, Lubbock, USA** – Bill Atkinson M.D.; **Klinikum Kreuzschwestern Wels, Wels, Austria** – Alfred Bacherer M.D.; **University of South Alabama, Mobile, USA** – Susan L. Baker M.D.; **Hôpital Arnaud de Villeneuve, Montpellier, France** – Laetitia Begue M.D.; **CHU Angers, Angers, France** – Florence Biquard M.D., **Regional Perinatal Group, Willis-Kinghton Health System, Shreveport, Louisiana, USA** – Christian Briery, M.D.; **Fetal Medicine Unit, Central Manchester University Hospitals NHS Foundation Trust, UK** – Phil Bullen M.D.; **Fundació Hospital Asil de Granollers, Barcelona, Spain** – Maria José Canto Rivera M.D.; **CHR Citadelle, CHU Liège, Belgium** – Frederic Chantraine M.D.; **Hull and East Yorkshire Hospitals NHS Trust, Hull, UK** – Anne Marie Coady M.D.; **Limoges Regional University Hospitals, Limoges, France** – Perrine Coste-Mazeau M.D.; **Hospital Universitari Dexeus, Barcelona, Spain** – Mónica Echevarria Telleria M.D.; **Hospital 12 de Octubre-IMAS 12, Madrid, Spain** – Alberto Galindo Izquierdo M.D.; **University Hospital Cologne, Cologne, Germany** – Ingo Gottschalk M.D.; **Piñon Perinatal, Albuquerque, USA** – Timothy J Hurley M.D.; **The John Radcliffe Hospital Oxford, Oxford, UK** – Lawrence Impey M.D.; **UZ Antwerpen, Antwerpen, Belgium** – Yves Jacquemyn M.D.; **University of Texas Medical Branch, Galveston, Texas, USA** – Sangeeta Jain M.D.; **Leiden University Medical Center, Leiden, The Netherlands** – Frans JCM Klumper M.D.; **Sheba Medical Center, Tel Hashomer, Israel** – Shlomo Lipitz M.D.; **Hospital Universitari Mutua de Terrassa, Barcelona, Spain** – Eva López Quesada M.D.;

Hospital Clínico Universitario Virgen de la Arrixaca-IMIB, Murcia – Juan Luis Delgado Marin M.D.; **Hospices Civils de Lyon, Lyon, France** – Jérome Massardier, M.D.; **Center for Assisted Reproduction and Prenatal Diagnosis, Minden, Germany** – Ralf Menkhaus M.D.; **Complejo Hospitalario de Navarra, Pamplona, Spain** – Ana Modroño Blanco M.D.; **University Hospital Jean Minjoz, Besançon, France** – Nicolas Mottet M.D.; **University Hospital Nîmes, Nîmes, France** – Eve Mousty M.D.; **Bambino Gesù Children's Hospital, Rome, Italy** – Antonella Nahom M.D.; **OLV van Lourdes Ziekenhuis, Waregem, Belgium** – Monica Notermans M.D.; **Maastricht UMC, Maasticht, The Netherlands** – Jos Offermans M.D.; **Hospital de la Santa Creu I Sant Pau, Barcelona, Spain** – Juan Parra Roca M.D.; **Stoke Mandeville Hospital, Aylesbury, UK** – Aparna Reddy M.D.; **Hospital Universitario Arnau de Vilanova, Lleida, Spain** – Ricardo Rosell Polo M.D.; **The Leeds Teaching Hospital, Leeds, UK** – Colete Sparey M.D.; **Hospital Universitario Miguel Servet, Zaragoza, Spain** – Mauricio Tajada Duaso M.D.; **CHRU Lille, Lille, France** – Pascal Vaast M.D.; **Ziekenhuis Geel, Geel, Belgium** – Ellen Vercammen M.D.; **University of Milano-Biocca, Monza, Italy** – Patrizia Vergani M.D.

Click to go to top of document: table of contents

Figure S1: Characteristics and outcomes in eligible non-participants to the study.

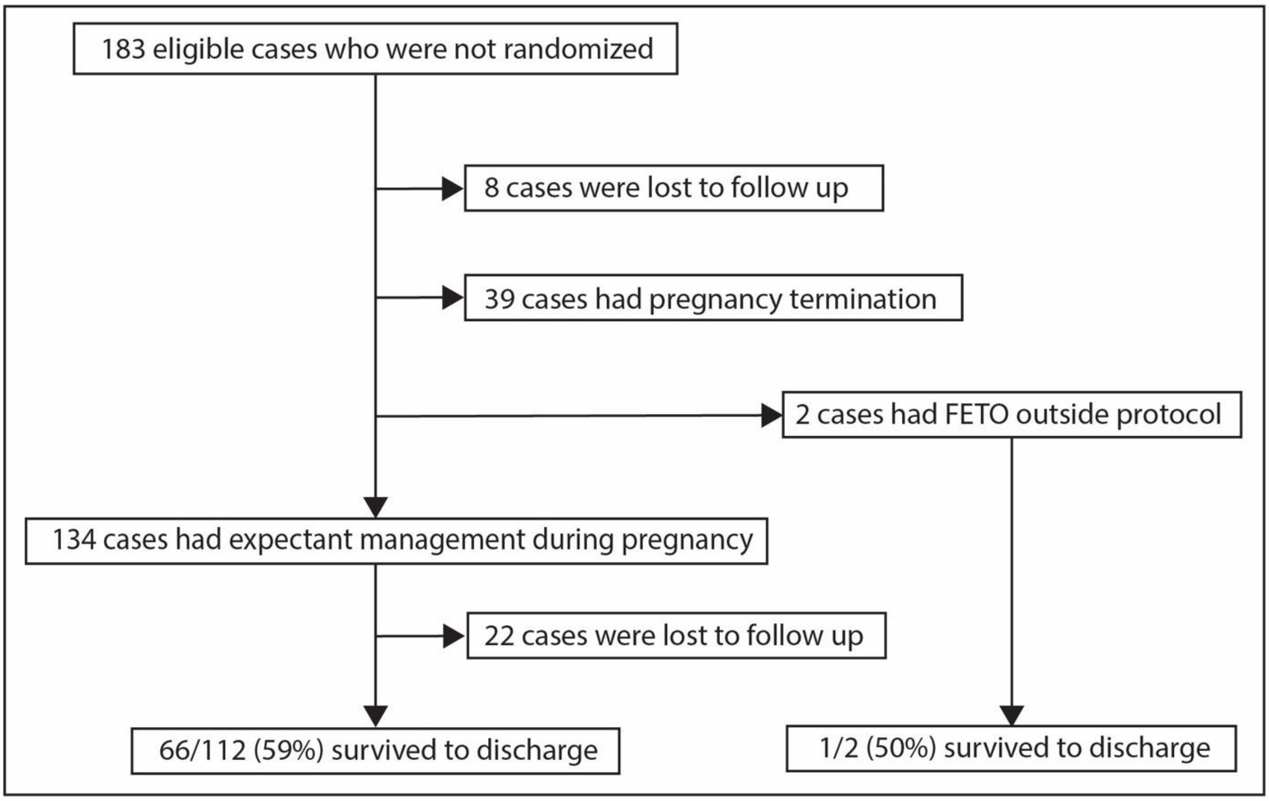

	n	O/E LHR	Liver herniation	Lost to follow up	Survival to discharge from NICU
Expectant management	134	31.0 (29.0-34.9)	106/134 (79%)	22/134 (16%)	66/112 (59%)
FETO	2	30.6 (27.0; 34.2)	2/2 (100%)	0/2 (0%)	1/2 (50%)

Continuous variables are reported as medians and interquartile ranges and categorical variables as absolute numbers and percentages.

[Click to go to top of document: table of contents]

Supplementary Table S1: Alphabetical list of FETO and their collaborating neonatal management centers

FETO centers	Neonatal management centers
Hospital Clinic, University of Barcelona, Barcelona, Spain	Hospital San Joan de Deu, Barcelona, Spain<br>Gregorio Maranon Hospital, Madrid, Spain<br>CHRU Montpellier, France
Universitätsklinikum Bonn, Germany	Universitätsklinikum Bonn, Germany<br>Klinikum Augsburg, Germany<br>University Hospital Mannheim, Germany<br>Johannes-Wessling-Klinikum, Minden, Germany
Mater Mothers' Hospital, Brisbane, Australia	Mater Mothers' Hospital, Brisbane, Australia<br>Women's and Children's Hospital, Adelaide, Australia<br>The Royal Women's Hospital and The Royal Children's Hospital, Melbourne, Australia
Hôpital Antoine Béclerè, Clamart, France	Hôpital Bicêtre, Paris, France<br>CHU Amiens, France<br>CHU Caen, France<br>CHU de Bordeaux, France<br>CHU Clermont-Ferrand, France<br>CHU Rennes, France<br>CHU de Dijon, France<br>CHRU Lille, France<br>CHU Nancy, France<br>Hôpital Trousseau, Paris, France<br>Hôpital Robert Debré, Paris, France<br>CHU Saint Etienne, France<br>Hôpital Bretonneau, CHU Tours, France
Baylor College of Medicine/Texas Children's Hospital, Houston, TX, USA	Texas Children's Hospital, Houston, TX, USA<br>University of Michigan Hospital, Ann Arbor, MI, USA<br>Banner Health – University Center Phoenix, Phoenix, AZ, USA
University of Texas Health Science Center / Children's Memorial Hermann, Houston, TX, USA	University of Texas Health Science Center / Children's Memorial Hermann, Houston, TX, USA
University Hospitals Leuven, Belgium	University Hospitals Leuven, Belgium<br>Ospedali Riuniti di Bergamo, Bergamo, Italy<br>UCL St Luc, Brussels , Belgium
	Medical University Hospital, Gdansk, Poland<br>LKH-Universitäts Klinikum Graz, Austria<br>Universitätsklinik Hamburg-Eppendorf, Germany<br>Shaare Zedek Medical Center, Jerusalem, Israel<br>Medical University Hospital, Krakow, Poland<br>Centre Hospitalier Universitaire Vaudois, Lausanne, Switzerland<br>Landes Frauen und Kinderklinik, Linz, Austria<br>Institute Polish Mothers Memorial Hospital, Lodz, Poland<br>Neue Frauenklinik, Luzern, Switzerland

	Radboudumc/Amalia Children's Hospital Nijmegen, The Netherlands<br>Clinical Hospital of Padua, Italy<br>The Institute for the Care for Mother and Child, Prague, Czech Republic<br>Erasmus MC, Rotterdam, The Netherlands<br>National Center for Fetal Medicine, Trondheim, Norway<br>Princess Anna Mazowiecka University Hospital, Warsaw, Poland<br>University Hospital Zurich, Switzerland
King's College Hospital, London, UK	King's College Hospital, London, UK<br>Leeds Teaching Hospitals NHS Trust, Leeds, UK<br>John Radcliffe Hospital, Oxford, UK<br>Royal London Hospital, UK<br>Central Manchester University Hospital, UK<br>St.George's Hospital, London, UK<br>Chelsea and Westminster, London, UK
Ospedale Maggiore Policlinico, Milano, Italy	Ospedale Maggiore Policlinico, Milano, Italy<br>Azienda Ospedaliero-Universitaria di Parma, Italy
Hôpital Necker – Enfants Malades, Paris, France	Hôpital Necker – Enfants Malades, Paris, Paris
Ospedale Pediatrico Bambino Gesù, Rome, Italy	Ospedale Pediatrico Bambino Gesù, Rome, Italy
1st Department of Obstetrics and Gynecology, Medical University of Warsaw, Poland	1st Department of Obstetrics and Gynecology, Medical University of Warsaw, Poland

Click to go to top of document: table of contents

Supplementary Table S2: List of primary, secondary and exploratory outcome measures, and safety endpoints prospectively collected via the electronic record forms

**Primary outcome**	**Definition**
Survival to discharge	Alive at discharge from NICU
**Co-primary outcome**	
Supplemental oxygen at 6 months of age	Any supplemental oxygen administration
**Secondary outcomes**	
Change in observed / expected LHR from baseline	(value before balloon removal- baseline value / baseline value)*100 (%)
Change in MRI O/E total lung volume from baseline	(value before balloon removal- baseline value / baseline value)*100 (%)
Grading of oxygen dependency according to Jobe and Bancalari[1]	Measured<br>- at d56 postnatal age or discharge, whichever comes first, for infants born $\geq$32w<br>- at 36w postmenstrual age or discharge, whichever comes first, for infants born <32w<br>Categorized as:<br>- Mild BPD: FiO2 0.21<br>- Moderate BPD: FiO2 0.22-0.29<br>- Severe BPD: FiO2 >0.30, and/or CPAP and/or mechanical ventilation
Pulmonary hypertension	Based on evidence on cardiac ultrasound of predominant unidirectional right to left shunt.
ECMO	Use of ECMO
Length of stay in NICU	Number of days from birth until discharge from NICU
Days of ventilatory support	Number of days of ventilatory support: mechanical ventilation, CPAP, optiflow. Low flow oxygen was not considered ventilatory support
Periventricular leukomalacia	As diagnosed by postnatal center using local criteria
Neonatal sepsis	As diagnosed by postnatal center using local criteria
Intraventricular hemorrhage (>grade III)	As diagnosed by postnatal center using local criteria
Retinopathy of prematurity	As diagnosed by postnatal center using local criteria
Days until full enteral feeding	Number of days from birth until full enteral feeding was established
Gastro-esophageal reflux	Above 1/3 of the esophagus on clinically indicated radiologic study
Day of postnatal surgery	Number of days from birth until the day of postnatal surgery
Use of patch	Use of patch at postnatal surgery
Defect size according to CDH study group[2]	A: small defect, with muscular edges all around<br>B: <50% chest wall involvement<br>C: > 50% chest wall involvement<br>D: total agenesis of diaphragm
Date of postnatal death	Date
Survival to 6 months of age	Survival at 6 months of age
**Exploratory outcomes**	
Gestational age at FETO and balloon removal	Gestational age in weeks and days
Successful placement of balloon	Balloon positioned between carina and vocal cords
Anesthesia during FETO and balloon removal	Local / neuraxial / general

Chorioamniotic membrane separation	Echolucent space between membranes and uterine wall evidenced on ultrasound examination
Spontaneous deflation of balloon	No balloon visible inside the fetal trachea at follow up ultrasound or at the time of fetoscopic removal
Polyhydramnios after FETO	Presence of a deepest vertical pocket of ≥ 8cm on ultrasound, diagnosed at any time point after FETO procedure
Gestational age at balloon removal	Gestational age in weeks and days
Emergency balloon removal	Balloon removal prior to scheduled date because of threatened preterm birth
Method of balloon removal	Fetoscopy / ultrasound guided puncture / postnatal
Interval balloon removal and delivery <24hrs	Duration between balloon removal and birth is less than 24 hours
Preterm prelabor rupture of membranes (PPROM) < 37 wks	Presence of ruptured membranes prior to 37 weeks gestational age
PPROM < 34 wks	Presence of ruptured membranes prior to 34 weeks gestational age
Gestational age at PPROM	Gestational age at membrane rupture, in weeks and days
Placental abruption	Occurrence of placental abruption
Gestational age at birth	Gestational age at birth, in weeks and days
Gestational age at birth < 32+0 wks	Birth < 32+0 wks
Gestational age at birth < 34+0 wks	Birth <34+0 wks
Gestational age at birth ≥ 37+0 wks	Birth ≥ 37+0 wks
Live born	Alive at birth
Birthweight	Birthweight in grams
Bronchopulmonary dysplasia according to Jobe and Bancalari[1]	Need for supplemental oxygen FiO2 >0.21 for at least 28 days. This is assessed at:<br>- >28 days but <56 days postnatal age or discharge, whichever comes first; for infants born ≥ 32 weeks<br>- 36 weeks postmenstrual age or discharge, whichever comes first, for infants born < 32 weeks
Necrotizing enterocolitis	Diagnosed by postnatal center using local criteria
Tracheomalacia	Diagnosed by postnatal center using local criteria
Survival to 28 days	Alive at day 28 after birth
Survival to 56 days	Alive at day 56 after birth
**Exploratory safety endpoints**	
IUFD < 24hrs after FETO	Occurrence of intrauterine fetal death within 24hrs after FETO procedure
IUFD at any point in pregnancy	Occurrence of intrauterine fetal death at any time point during pregnancy
Placental abruption < 24hrs after FETO	Occurrence of placental abruption within 24hrs after FETO procedure
Placental abruption at any point in pregnancy	Occurrence of placental abruption at any time point during pregnancy
Balloon removal issues	Any deviation from the normal course, or problem with balloon removal
Neonatal death due to failure of balloon removal	Neonatal death due to failure of balloon removal
Tracheomalacia	Diagnosed by postnatal center using local criteria
PPROM <37 wks	Presence of ruptured membranes prior to 37 weeks gestational age
Delivery <37 wks	Birth < 37+0 wks
Neonatal death <28 days	Neonatal death in the first 27 days
Death between 28 days and 6 months	Death between 28 days and 6 months after birth

Perinatal asphyxia (umbilical pH <7.00)	Umbilical artery pH <7.00
ECMO	Use of ECMO
Bronchopulmonary dysplasia according to Jobe and Bancalari[1] in survivors to discharge	Need for supplemental oxygen FiO2 >0.21 for at least 28 days. This is assessed at:<br>- >28 days but <56 days postnatal age or discharge, whichever comes first; for infants born ≥ 32 weeks<br>- 36 weeks postmenstrual age or discharge, whichever comes first, for infants born < 32 weeks
Pulmonary hypertension in survivors to discharge	Based on evidence on cardiac ultrasound of predominant unidirectional right to left shunt.
Periventricular leukomalacia in survivors to discharge	As diagnosed by postnatal center using local criteria
Neonatal sepsis in survivors to discharge	As diagnosed by postnatal center using local criteria
Intraventricular hemorrhage > grade III in survivors to discharge	As diagnosed by postnatal center using local criteria
Retinopathy of prematurity in survivors to discharge	As diagnosed by postnatal center using local criteria
Polyhydramnios after FETO	Presence of a deepest vertical pocket of ≥ 8cm on ultrasound, diagnosed at any time point after FETO procedure
Chorioamniotic membrane separation	Echolucent space between membranes and uterine wall evidenced on ultrasound examination
Any additional adverse event (free text field)	

[1]*Jobe AH, Bancalari E. Bronchopulmonary dysplasia. Am J Respir Crit Care Med. 2001 Jun;163(7):1723-9.*
[2]*Tsao K, Lally KP. The congenital diaphragmatic hernia study group: a voluntary international registry. Semin Pediatr Surg 2008;17:90-7.*

Click to go to top of document: table of contents

Supplementary Table S3: Associated anomalies diagnosed after randomization

Diagnosis	Time of diagnosis	Group	Included in per-protocol analysis (Yes/No)
Mutation filamin A gene	Postnatal	FETO	No
Mosaic trisomy 2	Postnatal	FETO	Yes
Duplication chromosome 8	Postnatal	FETO	Yes
Goldenhar	Postnatal	FETO	Yes
Simpson Golabi Behmel	Postnatal	FETO	No
Hypoplastic left heart syndrome	Postnatal	FETO	Yes
Tetrasomy 12p	Prenatal	Expectant	No
Tetrasomy 12p	Postnatal*	Expectant	Yes*
Fryns syndrome	Postnatal	Expectant	No

Summary of severe structural, genetic or syndromic diagnoses that were made **after** randomization. Inclusion into the per-protocol analysis was decided by the DMSC in consultation with two geneticists. 'Postnatal' (column 2) indicates the diagnosis was made during the postnatal period, though prior to discharge from the NICU. * In this infant, the diagnosis of tetrasomy 12p was made at one year of age, because of developmental delay. Because of the late diagnosis, the case was included by the DSMC in the per protocol analysis.

Click to go to top of document: table of contents

Supplementary Table S4: Operative outcomes in patients in the FETO arm.

Characteristic	
**FETO procedure (n=98)**	
FETO not performed – no. (%)	7/98 (7)
Failed placement of balloon – no. (%)	1/98 (1)
Successful placement of balloon – no. (%)	90/98 (92)
**FETO attempted (n=91)**	
Gestational age at FETO – wk	30.4 (30.1 – 30.8)
Anesthesia: local – no. (%)	35/91 (38)
Anesthesia: neuraxial block – no. (%)	56/91 (62)
**Balloon removal procedure (n=90)**	
Gestational age at balloon removal - wk	34.0 (33.2 – 34.3)
Spontaneous deflation no removal – no. (%)	1 (1)
Emergency balloon removal – no. (%)*	35 (39)
Gestational age - wk	33.0 (32.4 – 33.6)
Method: fetoscopic– no. (%)	24/35 (69)
Method: ultrasound guided puncture – no. (%)	4/35 (11)
Postnatal – no. (%)	7/35 (20)
Elective balloon removal – no. (%)	54 (60)[a]
Gestational age - wk	34.3 (34.1 – 34.6)[a]
Method: fetoscopic – no. (%)	53/54 (98)[a]
Method: ultrasound guided puncture – no. (%)	1/54 (2)
**Interval between balloon insertion and removal**	
Duration of tracheal occlusion – days[#]	24 (19 – 28)
Change in O/E LHR from baseline – %	32 (1 – 66)[b]
**Interval between balloon removal and delivery**	
< 24 hours – no. (%)	17/90 (19)

Continuous variables are reported as medians and interquartile ranges and categorical variables as absolute numbers and percentages. Abbreviations: FETO: fetoscopic endoluminal tracheal occlusion; O/E LHR: observed/expected lung-to-head ratio. [a] including two fetuses where the balloon was deflated; [b] six missing values. All exploratory outcomes except for change in O/E LHR from baseline.

* Reasons given were one or more of the following: prelabor membrane rupture (n=19), preterm labor (n=18), polyhydramnios (n=3), vaginal bleeding (n=2), large chorioamniotic membrane separation (n=1), delivery because of fetal growth restriction (n=1) or severe preeclampsia (n=1).
[#] excluding three cases with spontaneous deflations

Click to go to top of document: table of contents

Supplementary Table S5: Secondary neonatal outcomes in survivors at discharge

Outcome	FETO (n=62)	Expectant (n=49)
Days to neonatal repair of defect	3 (2-6)	2 (2-4)
ECMO days in survivors receiving ECMO (n=16)	6 (5-8)	7 (6-8)
Days of ventilatory support	25 (13-43)	28 (14-41)
Days to full enteral feeding	28 (17-40)	27 (18-40)
Bronchopulmonary dysplasia – no. (%)°	41/62 (66)	32/49 (65)
Mild	19/41 (46)	18/32 (56)
Moderate	11/41 (27)	7/32 (22)
Severe	11/41 (27)	7/32 (22)
Pulmonary hypertension – no. (%)	46/62 (74)	33/49 (67)
Periventricular leukomalacia – no. (%)	3/62 (5)[a]	1/49 (2)[b]
Sepsis – no. (%)	21/62 (34)	17/49 (35)
Intraventricular hemorrhage > grade III – no. (%)	0/62 (0)	0/49 (0)
Retinopathy of prematurity > grade 3 – no. (%)	0/62 (0)	0/49 (0)
Necrotizing enterocolitis – no. (%)°	2/62 (3)	0/49 (0)
Gastroesophageal reflux – no. (%)[c]	32/60 (53)	19/39 (49)
NICU days	49 (29-80)	46 (29-72)

Results are reported as absolute numbers and percentages (categorical variables) and or as medians and interquartile ranges (continuous variables) [a] all grade 1; [b] grade 3; [c] 12 missing values. °exploratory outcomes

[Click to go to top of document: table of contents]

Supplementary Table S6: Results of all interim analyses

						One-sided analysis[c]		Two-sided analysis[c]	
**Analysis stage**	**Date**	**Target N/arm**	**FETO, n (%)**	**Expectant management, n (%)**	**Difference between percentages**	**p-value**	**Alpha[d]**	**p-value**	**Alpha[d]**
Interim 1	Oct 15	40	24/40 (60.0)	18/40 (45.0)	15.0	0.0871	0.0005	0.1742	0.0010
Interim 2	Feb 17	59	37/59 (62.7)	28/59 (47.5)	15.3	0.0460	0.0036	0.0919	0.0072
Interim 3[a]	Oct 17	69	45/70 (64.3)	33/68 (48.5)	15.8	0.0294	0.0064	0.0587	0.0128
Interim 4	Sep 18	79	51/79 (64.6)	38/79 (48.1)	16.5	0.0173	0.0099	0.0345	0.0198
Interim 5[b]	Feb 19	89	57/89 (64.0)	43/88 (48.9)	15.2	0.0197	0.0140	0.0393	0.0280
Final	Mar 20	98	62/98 (63.3)	49/98 (50.0)	13.3	0.0293	0.0186	0.0587	0.0372

[a] Due to a randomization error, there were 70 patients in FETO arm and 68 in the expectant management arm

[b] One outcome in expectant management arm not available at time of analysis

[c] The statistical design and analysis was done using a one-sided test (while controlling the overall alpha at 2.5%) focusing on superiority of FETO. We have post hoc added the two-sided results (overall alpha 5%), which equals twice the one-sided results if the observed effect favored FETO. With a two-sided approach, the direction of the observed effect must be checked.

[d] The alpha level at each analysis stage is defined using the O'Brien-Fleming method in order to control the overall alpha at 2.5% (one-sided) or 5% (two-sided).

Observed survival rates for both study arms

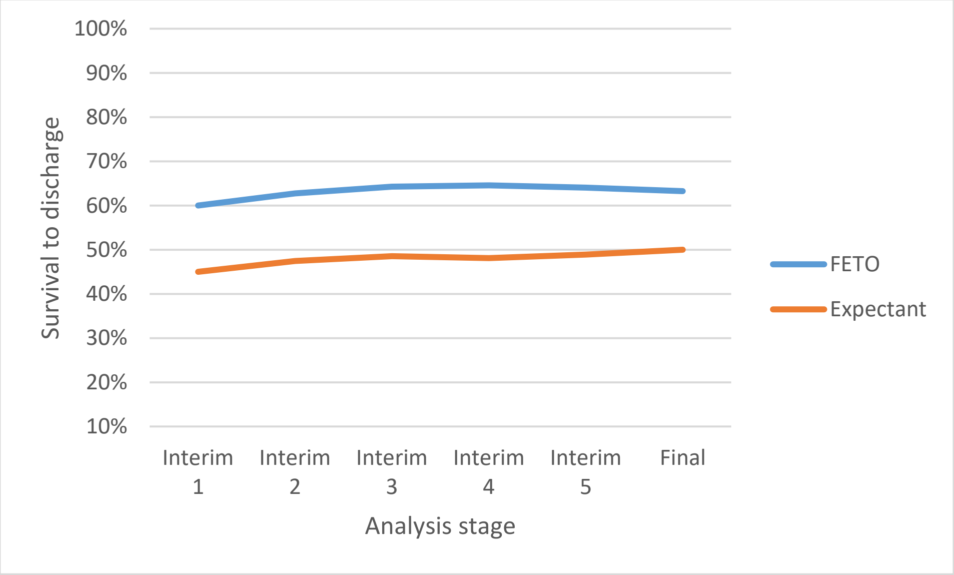

Click to go to top of document: table of contents

Supplementary Table S7: Case load for postnatal management centers two years prior to their first trial patient.

Center	Y1	Y2
Hospital San Joan de Deu, Barcelona	14	14
Gregorio Maranon Hospital, Madrid	7	8
CHRU Montpellier, France	4	3
Universitätsklinikum Bonn	14	11
University Hospital Mannheim	46	59
Mater Mothers' Hospital, Brisbane	3	2
Women's and Children's Hospital, Adelaide	2	8
The Royal Women's Hospital and The Royal Children's Hospital, Melbourne	4	13
Hôpital Bicêtre, Le Kremlin-Bicêtre	17	13
CHU Amiens	6	3
CHU Caen	2	2
CHU de Bordeaux	6	6
CHU Clermont-Ferrand	7	4
CHU Rennes	2	3
CHU de Dijon	5	4
CHRU Lille	13	16
CHU Nancy	3	1
Hôpital Trousseau, Paris	7	6
Hôpital Robert Debré, Paris	8	8
CHU Saint Etienne	1	2
Hôpital Bretonneau, CHU Tours	7	13
Texas Children's Hospital, Houston	14	24
University of Michigan Hospital, Ann Arbor	12	22
University of Texas Health Science Center/ Children's Memorial Hermann, Houston	11	3
University Hospitals Leuven	15	11
Saint Luc UCL, Brussels	4	2
Medical University Hospital, Gdansk	3	0
LKH-Universitäts Klinikum Graz	4	4
Universitätsklinik Hamburg-Eppendorf	1	1
Radboudumc/Amalia Children's Hospital Nijmegen	6	22
The Institute for the Care of Mother and Child, Prague	8	18
Erasmus MC, Rotterdam	11	14
National Center for Fetal Medicine, Trondheim	6	2
University Hospital Zurich	8	7
King's College Hospital, London	23	20
St.George's Hospital, London	10	7
Chelsea and Westminster, London	12	12
Ospedale Maggiore Policlinico, Milano	15	18

Hôpital Necker – Enfants Malades, Paris	9	11
Ospedale Pediatrico Bambino Gesù, Rome	16	17
1st Department of Obstetrics and Gynecology, Medical University of Warsaw	22	28

Seventeen neonatal management centers did not provide historical data.

Click to go to top of document: table of contents
